# Comparison between simultaneous bilateral total hip arthroplasty with and without drainage: A retrospective cohort study

**DOI:** 10.1097/MD.0000000000031134

**Published:** 2022-10-28

**Authors:** Min-Gwang Kim, Chae-Jin Im, Woo-Chul Jung, Taek-Rim Yoon, Kyung-Soon Park

**Affiliations:** a Center for Joint Disease, Department of Orthopedic Surgery, Chonnam National University Medical School and Hwasun Hospital, Hwasun-gun, Republic of Korea.

**Keywords:** blood loss, drainage, pain, simultaneous bilateral, total hip arthroplasty

## Abstract

Simultaneous bilateral total hip arthroplasty (SBTHA) is an effective procedure for patients with disease bilaterally. But there is concern about increased blood loss and complications of SBTHA than staged total hip arthroplasty (THA). This study aimed to evaluate the differences in the clinical outcomes and complication rate of SBTHA with drainage and without drainage for reducing the concerns. Between October 2015 and April 2019, a retrospective cohort study was conducted with modified minimally invasive 2-incision method and a consecutive series of 41 SBTHA performed with drainage (Group I) were compared to 37 SBTHA performed without drainage (Group II). It was assessed clinically and radiographically for a mean of 2.1 ± 0.8 years (range, 1.0-4.8 years). Postoperative hematologic values (Hgb loss, total blood loss, transfusion rate), pain susceptibility, functional outcome (Harris Hip Score, Western Ontario and McMaster Universities Osteoarthritis Index score) and complication were compared in the drained group and the non-drained group. Postoperative Hgb loss (I: 2163.2 ± 698.7 g, II: 1730.4 ± 572.5 g; *P* = .002), total blood loss (I: 1528.8 ± 421.7 mL, II: 1237.6 ± 325.9 mL; *P* = .001) and mean transfusion unit (I: 0.7 ± 1.0 IU, II: 0.1 ± 0.3 IU; *P* < .001) were significantly lower in the without drainage group than in the with drainage group. But the morphine equivalent (I: 132.7 ± 314.1 mg, II: 732.2 ± 591.5 mg; *P* < .001) was significantly larger in the without drainage group. No significant difference was found between the drainage group and without drainage group in Harris Hip Score and Western Ontario and McMaster Universities Osteoarthritis Index score at final follow-up. SBTHA without drainage can reduce postoperative blood loss and the requirement for transfusion without increasing other complication. But SBTHA without drainage is more painful method than SBTHA with drainage. Therefore, SBTHA without drainage will be a good option to reduce the burden on the patient by reducing postoperative bleeding if it can control pain well after surgery. **III**, Retrospective case-control study.

## 1. Introduction

The number of patients undergoing total hip arthroplasty (THA) worldwide is increasing every year due to an increase in the prevalence of osteoarthritis (OA) of the hip joint and osteonecrosis of the femoral head (ONFH), among other causes. Bilateral hip joint disease is so frequent that it can occur in up to 42% of patients with OA, those undergoing unilateral THA with bilateral disease if they had Grade 3 and 4 radiographic changes and clinically displayed moderate or severe symptoms had a 97% chance of OA progression, eventually required contralateral THA.^[1]^

Rheumatoid arthritis, slipped capital femoral epiphysis, developmental dysplasia of the hip, and ONFH are also less common causes, but mainly affect both hip joints. These patients are required to undergo a total bilateral hip arthroplasty, sequentially or simultaneously.

Simultaneous bilateral total hip arthroplasty (SBTHA) has many advantages like a shorter hospitalization period, faster rehabilitation, high patient satisfaction, and lower total hospital cost.^[2–5]^ Thus, cases of SBTHA have been recently increasing. However, SBTHA causes more blood loss than staged THA, and patients might require allogenic blood transfusion.^[6,7]^ It is well known that allogenic blood transfusion in patients undergoing THA can cause complications, such as infection, delay of rehabilitation, prolonged hospital stay, and increase hospital cost.^[8]^

Multiple efforts have been made for reducing the requirement for allogenic blood transfusion after THA and not using drainage was considered as one of many efforts. There is still controversy over not using drainage after THA; however, not using drainage after THA has been reported to reduce blood loss. There are many reports demonstrating that unilateral THA without drainage can reduce blood loss, and there is no difference in the risk of complications between THA performed with and without drainage.^[9–11]^ However, there is little report regarding SBTHA without drainage.^[12]^ Thus, in this study, we aimed to evaluate the differences in the outcomes of SBTHA with drainage and without drainage and identify any other associated complication.

## 2. Material and Methods

### 2.1. Patients

We performed a retrospective study on patients who underwent SBTHA for OA of the hip joint or ONFH with a follow-up of at least 1-year at our institution. Between October 2015 and April 2019, 88 patients (176 hips) were treated with modified minimally invasive 2-incision method by 2 surgeons. All THAs were done by 2 experienced high volume (>300 hip surgeries per year) hip surgeons.

**Figure 1.**
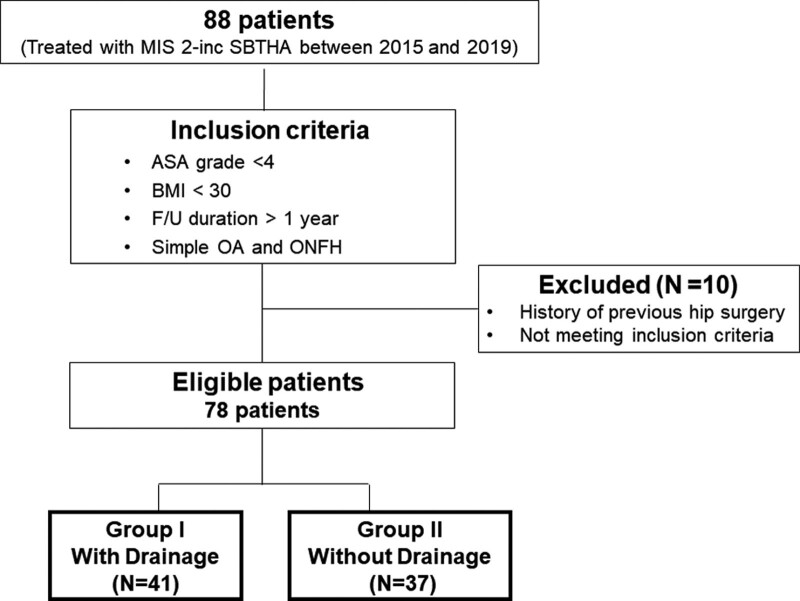
Flow diagram of patient selection.

The inclusion criteria included an American Society of Anesthesiologists (ASA)^[13]^ grade < 4, body mass index < 30, completed ≥ 1-year of follow-up, and presence of disease such as OA and ONFH to be treated with simple THA. The exclusion criteria included history of previous hip surgery, follow-up loss during the study period, and severe unilateral or bilateral deformities such as septic hip sequelae, developmental dysplasia of the hip sequelae, posttraumatic OA or AVN. Patients with severe deformities of the hip joint and adhesion of the surgical site due to a previous surgery were excluded for easy comparison as this could increase the amount of bleeding during surgery. Finally, a total of 78 patients (156 hips) were eligible for inclusion in the study (46 men, 32 women). 41 patients (20 men, 21 women) underwent SBTHA with drainage (Group I) and 37 patients (26 men, 11 women) underwent SBTHA without drainage (Group II) (Fig. 1).

### 2.2. Ethics approval and consent to participate

The patients/legal guardians signed written informed consent to participate in the study and agreed to use their images for publication of this article. In this study, all methods were performed in accordance with the relevant guidelines and regulations. All rights of the patients were protected against any kind of disadvantage and individual matters. This study was conducted with the approval of the institutional review board.

### 2.3. Surgical procedure

All operations were performed using modified minimally invasive 2-incision SBTHA in a lateral position, which method uses part of the Watson-Jones approach for anterior incision and part of the Moore posterior approach for posterior incision.^[14]^ All patients, except those with thromboembolic tendency, received intravenous tranexamic acid (TXA) (15 mg/kg) prior to skin incision of the first hip and received intra-articular TXA (15 mg/kg) within safety dose before wound closure of each hip. Femoral implanting using cementless tapered wedge stem was followed by Acetabular implanting using cementless acetabular cup in a press-fit manner.

In group I, before the subcutaneous layer was closed, drainage was placed under the fascia. The patients were treated by using positive pressure for 24 hours postoperatively and after 24 hours, the negative pressure was maintained until the insertion was removed. The volume of drained blood was recorded daily and the drain tube was removed if the volume was <100 mL for a day.^[15]^ In Group II, the drainage was not inserted, so the wound was covered with water-proof wound dressing material also used in group I after subcutaneous layer was closed.

### 2.4. Postoperative management

After the surgery, hematologic values were checked preoperatively, immediately and on 1, 3, and 5 days after surgery. If the hemoglobin level was <8.0 g/dL, patients received red blood cell transfusion and the average transfusion unit (IU) was calculated.^[16]^ The volume of packed RBC was 320 mL per unit. Intermittent pneumatic compression device are used to help prevent deep venous thromboembolism. Intravenous patient-controlled analgesia was used as a postoperative routine for all patients. Nevertheless, if the patient complained of pain, visual analogue scale pain score 5 or higher, oral or parenteral opioids like tramadol IV, pethidine IV, or fentanyl transdermal patch were prescribed to control pain during hospitalization depending on the circumstances. The same rehabilitation protocol was used for both groups. On the first postoperative day, quadriceps strengthening exercises and ankle dorsiflexion exercise were initiated and ambulation with aid was recommended on the third postoperative day. If the patient’s general condition permitted, all patients were scheduled to be discharged within 7 days after surgery.

### 2.5. Clinical outcome measurements

All hips were reviewed by an orthopedic surgeon who had not been involved with the original surgical procedure, and the data were entered into a computerized record.

Estimated blood volume was calculated by the formula described by Nadler et al^[17]^ as follows:

Blood volume(ml) = (k1 × height(m)^3^ + k2 × weight(kg) + k3) × 1000

(For men, k1 = 0.3669, k2 = 0.03219, k3 = 0.6041; for women, k1 = 0.3561, k2 = 0.03308, k3 = 0.1833).

Based on the Hgb balance, the blood loss was calculated according to the formula described by Good et al^[18]^ as follows:

Hgb loss(g) = (BV × (Hgb_pre_ − Hgb_post_))×0.001 + Hbt

Total blood loss = 1000 × Hgb loss/Hb_pre_

(Hgb loss(g); the amount of Hb lost, Hgb_pre_(g/L); the Hgb concentration before surgery, Hgb_post_ (g/L)l; the lowest Hgb concentration after surgery, Hbt (g); the total amount of allogeneic Hb transfused).

We evaluated postoperative pain susceptibility using morphine equivalent conversion formation for quantitative comparison.^[19]^ Functional outcome scoring such as Harris Hip Score, The Western Ontario and McMaster Universities Osteoarthritis Index were collected from preoperative visit to final follow up. Lastly occurrence of wound complications (skin necrosis, wound dehiscence, superficial infection), mechanical complications (intraoperative or postoperative periprosthetic fracture) and medical complications (deep vein thrombosis, pulmonary thromboembolism) during hospitalization period and deep infection during follow-up period were assessed.

### 2.6. Statistical analysis

Statistical analyses were performed using SPSS statistical software system (IBM Corp. Released 2016. IBM SPSS Statistics for Windows, Version 24.0. Armonk, NY: IBM Corp.). Differences in normally distributed variables were analyzed using the independent *t* tests. Differences in categorical variables between the groups were used to analyze using Chi-squared or Fisher’s exact tests. A significance level of ≤0.05 was used for all statistical tests.

## 3. Results

The patients’ demographic information is summarized in Table 1. There were no differences in patient demographic details such as age, sex, body mass index (kg/m^2^), ASA status between the 2 groups. The patients were followed clinically and radiographically for a mean of 2.0 years (range, 1.0-3.8 years) in group I and 2.3 years (range, 1.0-4.8 years) in group II.

**Table 1:** Demographic details, anesthesia risk and preoperative diagnosis of patients.

Parameter	With drainage (N = 41)	Without drainage (N = 37)	*P* value
Male/female†	20/21 (49%/51%)	26/11 (70%/30%)	.054
Age (yrs)*	51.8 ± 11.2 (range 23-77)	50.4 ± 15.1 (range 25-76)	.645
Body mass index*	24.7 ± 4.0 (range 18.4-39.8)	24.2 ± 3.8 (range 18.5-35.3)	.566
ASA class†			.558
1	26 (63%)	22 (59%)	
2	15 (37%)	14 (38%)	
3	0 (0%)	1 (3%)	
**Preoperative diagnosis** †			.410
ONFH	24 (59%)	25 (67%)	
OA	17(41%)	12 (33%)	

* Independent *t* test.

† Pearson’s chi-square test. Data are presented as median ± standard deviation (range minimum result—maximum result) and also numbers of patients (percentage). The p-values reflect the results of inter-group comparisons, with *P* < .05 indicating significance.

ASA = American Society of Anesthesiologists, OA = osteoarthritis, ONFH = osteonecrosis of femoral head.

### 3.1. Hematologic values

**Figure 2.**
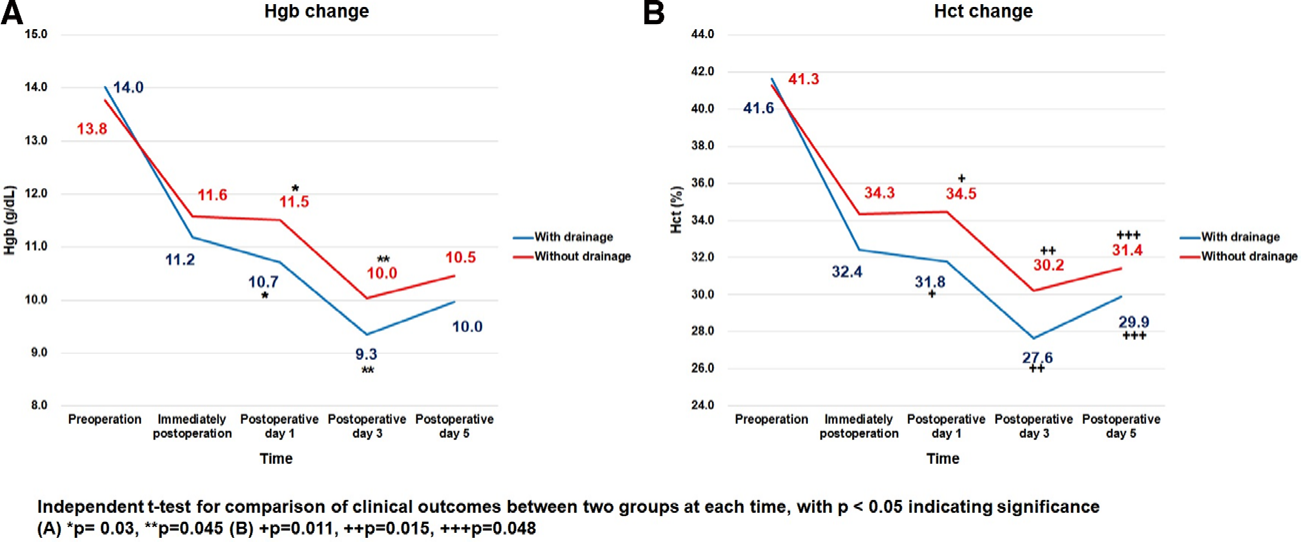
Change of hematologic values between 2 groups.

In this study, Figure 2 showed a decreasing trend of Hgb (g/dL) and Hct (%) immediately after surgery until 3 days after surgery and then recovered. The non-drainage group had higher Hgb and Hct levels than the drainage group on the first (*P* = .03 and *P* = .011) and third (*P* = .045 and *P* = .015) postoperative days with significant difference. Whereas there was significant difference between the drainage and non-drainage groups in only Hct levels (*P* = .048).

When these changes in the 2 groups were compared with the average of the preoperative and postoperative measures, there were significant differences between the preoperative and postoperative hematological values (Table 2).

**Table 2:** Clinical details and perioperative data of the 2 groups.

Parameter	With drainage (N = 41)	Without drainage (N = 37)	*P* value
Clinical outcome			
Estimated blood volume (mL)*	4139.9 ± 790.6	4406.2 ± 691.3	.119
Preoperative Hgb (g/dL)*	14 ± 1.6	13.8 ± 1.5	.474
** Lowest postoperative Hgb (g/dL**)*	**9.0 ± 1.6**	**9.9 ± 1.2**	**.006**
** Hgb loss (g**)*	**2163.2 ± 698.7**	**1730.4 ± 572.5**	**.002**
** Total blood loss (mL**)*	**1528.8 ± 421.7**	**1237.6 ± 325.9**	**.001**
Total drainage (mL)	769.4 ± 279.7		
** Number of blood recipient (%**)†	**18 (44%**)	**1 (3%**)	**<.001**
** Amount of transfusion (IU**)*	**0.7 ± 1.0**	**0.1 ± 0.3**	**<.001**
** Pain susceptibility (ME, mg**)*	**132.7 ± 314.1**	**732.2 ± 591.5**	**<.001**
Functional outcome			
Preoperative HHS*	54.5 ± 17.7	51.7 ± 17.5	.484
Preoperative WOMAC score*	52.4 ± 16.8	54.4 ± 18.9	.624
Last f/u HHS*	90.5 ± 10.6	93.2 ± 8.3	.208
Last f/u WOMAC score*	8.2 ± 6.3	6.0 ± 6.7	.151

Data are presented as median ± standard deviation and also numbers of patients (percentage). The *P* values reflect the results of inter-group comparisons, with *P* < .05 indicating significance.

Hgb = hemoglobin, HHS =Harris Hip Score, ME =morphine equivalent, WOMAC =Western Ontario McMaster Universities Osteoarthritis Index.

* Independent *t* test.

† Fisher’s exact test.

Postoperative Hgb loss (I: 2163.2 ± 698.7 g, II: 1730.4 ± 572.5 g; *P* = .002), total blood loss (I: 1528.8 ± 421.7 mL, II: 1237.6 ± 325.9 mL; *P* = .001) was significantly lower in Group II than in Group I; therefore, the transfusion requirements were significantly lower in Group II (0.1 ± 0.3 IU) than in Group I (0.7 ± 1.0 IU, *P* < .001). As a result, 18 of 41 patients (44%) in group I received blood transfusions, whereas only 1 of 37 patients (3%, *P* < .001) received blood transfusions in group II.

### 3.2. Pain susceptibility

The mean dose of morphine equivalent (mg) used was significantly larger in Group II (732.2 ± 591.5 mg) than in Group I (132.7 ± 314.1 mg, *P* < .001) (Table 2), which means that the patients in Group II felt more postoperative pain than the patients in Group I.

### 3.3. Functional outcome

The mean Harris Hip Score improved in Group I from 54.5 (±17.7) preoperatively to 90.5 (±10.6) postoperatively, and in Group II from 51.7 (±17.5) preoperatively to 93.2 (±8.3) postoperatively (*P* = .208). The mean Western Ontario and McMaster Universities Osteoarthritis Index was 52.4 (±16.8) in Group I and 54.4 (±18.9) in Group II. This improved to 8.2 (±6.3) in Group I and 6.0 (±6.7) in Group II (*P* = .151).

### 3.4. Complication

Perioperative complications of both groups are listed in Table 3. In Group I, 2 hips had an intraoperative fracture, and 1 hip had a postoperative fracture. Also, 6 hips had a prophylactic cerclage wire placement for reducing the risk of early periprosthetic fracture. In Group II, 2 hips had a postoperative fracture, while there were no intraoperative fractures. Also, 3 hips had a prophylactic cerclage wire placement. Superficial infection occurred in 1 hip only in Group I and responded to local wound care and antibiotics. Skin necrosis, wound dehiscence, deep infection and medical complications such as deep venous thrombosis, pulmonary thromboembolism was not detected in any group.

**Table 3:** Perioperative complications of both groups, by number and percentage.

Parameter	With drainage(N = 41, 82 hip)	Without drainage(N = 37, 74 hip)	*P* value
Wound complications			
Skin necrosis*	0 (0.0%)	0 (0.0%)	1
Wound dehiscence*	0 (0.0%)	0 (0.0%)	1
Superficial infection*	1 (1.2%)	0 (0.0%)	.344
Mechanical complications			
Periprosthetic fracture			
- Intraoperative*	2 (2.4%)	0 (0.0%)	.159
- Postoperative*	1 (1.2%)	2 (2.7%)	.504
Prophylactic wiring*	6 (7.3%)	3 (4.0%)	.386
Medical complications			
DVT*	0 (0.0%)	0 (0.0%)	1
PTE*	0 (0.0%)	0 (0.0%)	1
Deep infection*	0 (0.0%)	0 (0.0%)	1

* Fisher’s exact test. Data are presented as numbers of affected hip joint (percentage). The *P* values reflect the results of inter-group comparisons, with *P* < .05 indicating significance.

DVT = deep vein thrombosis, PTE = pulmonary thromboembolism.

## 4. Discussion

Regardless of the use of drainage, the 2 groups presented similar results in clinical outcomes such as functional score and complications at a mean follow up of 25 months. The most important finding of this study was disusing of drainage reduces loss of bleeding and minimizes the requirement of blood transfusions after SBTHA.

Unlike the current situation where the demand for SBTHA is increasing, there is little report about SBTHA without drainage. So this study was conducted with our expectation of the superiority of SBTHA without drainage. Blood loss was found to be less in the non-drainage group compared to that in the drainage group. In non-drainage group, this may be a consequence of the absence of blood loss into the drain and of none of the reduced effectiveness of topical TXA that was drained immediately with drainage. Many studies have described a positive correlation between non drainage and clinical outcomes after THA, which is consistent with the results of this study.

Other strategies have been attempted to minimize blood loss recently. Among them, TXA is in the spotlight in reducing perioperative blood loss in total joint arthroplasty, recently.^[20]^ Previous articles have demonstrated that a combined administration of TXA in THA is associated with a significantly reduced total blood loss and was safe for patients who received intravenous administration and topical application of TXA.^[21]^ Steven BP et al proved that it is safe to use in high risk patients with pro-thrombotic conditions. This study gives additional credence to the recent clinical guide on TXA administration in THA patients.^[22]^

**Figure 3.**
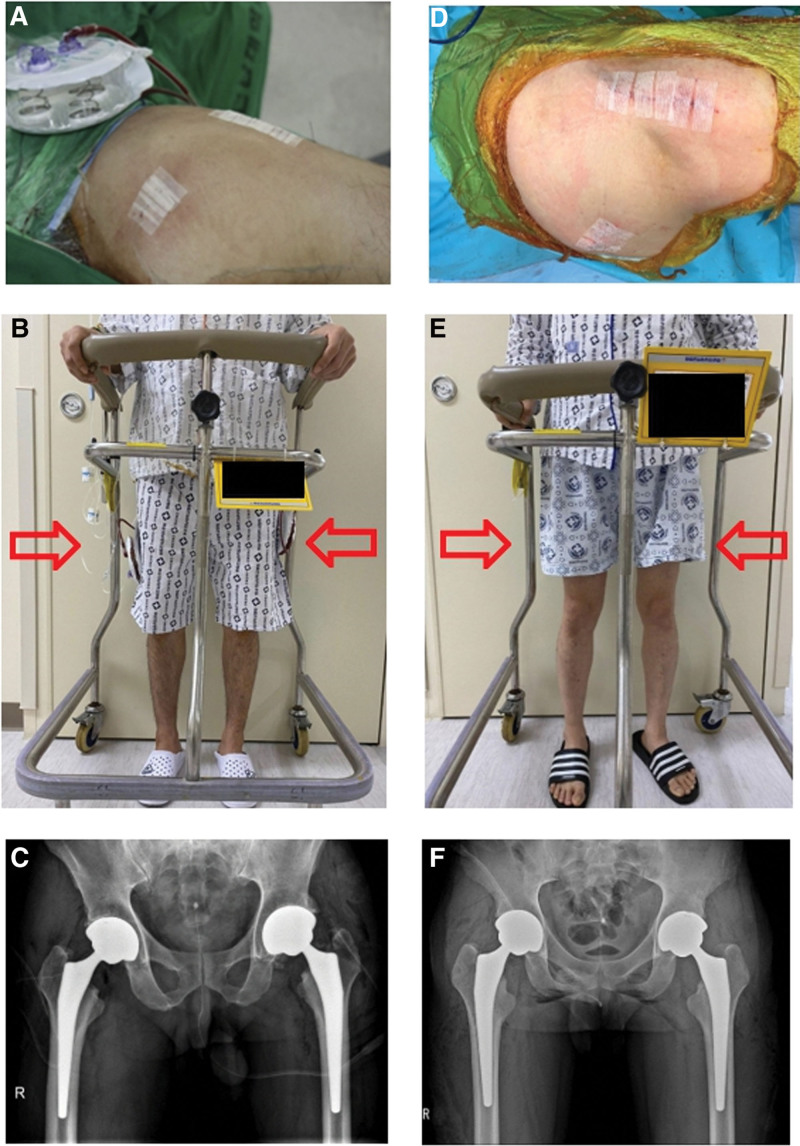
Overall comparison from intraoperative findings to during hospitalization between 2 groups. (A, B) The patients in group I has drainage line, making it difficult to walk after surgery, and the drainage line may pull out due to jamming while walking. (D, E) But the patients in group II has no drainage line, making it more convenient to walk after surgery than the patient with drainage, there was also no risk of disconnection of drainage line. The patient’s satisfaction after SBTHA without drainage is higher in our institution. (C, F) These 2 plain radiographs are pictures of each group immediately after surgery. SBTHA = simultaneous bilateral total hip arthroplasty.

The higher the blood loss, the higher the incidence of blood transfusions. Blood transfusion requirements tended to be higher for patients in the drainage group. Kim et al reported in a meta-analysis that allogenic blood transfusion is a significant risk factor for increasing the surgical site infection rate after total hip and knee joint arthroplasty.^[23]^ However, in our study, we found no correlation between allogenic blood transfusion and surgical site infection. Also when drainage is not performed, blood may leak through the wound, which disrupts wound healing and may cause a surgical site infection. But in our study, there were no wound problem and significant infection cases. Furthermore, the non-drainage group generally showed higher satisfaction with easier movement during dressing of operation site and ambulation because of no drainage line (Fig. 3).

Along with the positive aspects of without drainage, there were negative aspects as well. Higher morphine equivalent was derived in the patients without drainage, which means they felt more postoperative pain than the patients who underwent SBTHA with drainage. Rajesh and Prashant et al proved that the presence of drainage significantly reduces opioid consumption during the first 6 hours after total knee arthroplasty.^[24]^ Li S et al randomly divided the patients undergoing high tibial osteotomy into a drainage group and a no drainage group and the results showed that there was improvement of visual analogue scale pain score in the non-drainage group until postoperative 5 days.^[25]^ This is supported the fact that suction drainage can decrease immediate postoperative pain by draining hematoma production that results in increased pressure at the surgical site. But Park CW et al insisted that postoperative daily pain were not significantly different between the drainage and non-drainage groups undergoing SBTHA throughout the hospital admission.^[26]^ They recorded postoperative daily pain scales at least 3 times a day using the Numerical Rating Scale (NRS). The NRS is a subjective pain assessment method and it is a validated method for patients to evaluate the degree of pain. However, we postulate that differences would not have emerged in the study since NRS does not have an exact baseline for the introduction of pain control medication.

In this study, since TXA was used by same method in both groups, the effect could be excluded, but TXA also contributes to reducing postoperative pain. TXA is a synthetic derivative of the amino acid lysine and binds the lysine binding sites on plasminogen, interfering with plasminogen binding to fibrin. By inactivating plasmin, TXA can prevent hyperfibrinolysis. Additionally, the coagulation-fibrinolysis process has been identified at interconnecting with inflammatory cascade.^[27]^ Therefore TXA could play a exert an anti-inflammatory effect by inhibiting plasmin-mediated activation of complement, monocytes, and neutrophils recruitment to the implanted biomaterials, which may consequently minimize postoperative pain.^[28]^ Administration of TXA also helps to reduce postoperative pain by reducing intra-articular hemarthrosis.^[29]^ But JW Wurtz et al suggested an opposing opinion about role of TXA reducing postoperative pain. In this study, patients who received topical TXA reported higher mean 24-hour pain scores (*P* = .006) and requested opioid sooner (*P* = .033) compared to patients who did not receive TXA.^[30]^

Several limitations existed in this study. Firstly, this was a retrospective cohort study based on the database of a single institute. Secondly, the mean follow-up period was not long enough to evaluate the long-term clinical outcomes. Thirdly, patients who undergo SBTHA usually have good physical conditions such as ASA grade 1 and 2; therefore, the result remain unknown in less healthier populations. Lastly, further studies are needed on the effect of TXA on postoperative pain.

## 5. Conclusion

SBTHA without drainage can reduce postoperative blood loss and the requirement for transfusion without increasing other complication. But SBTHA without drainage is more painful method than SBTHA with drainage. Therefore, SBTHA without drainage will be a good option to reduce the burden on the patient by reducing postoperative bleeding if proper pain management is used.

## Acknowledgments

The authors thank all clinical researchers involved in the research we included in this article. This study was not supported by any company or grant.

## Author contributions

MG KIM: Collection and analysis of data and writing manuscript. CJ IM, WC JUNG: Collection and analysis of data. TR YOON: Make concepts and design of study. KS PARK: Make concepts and design of study. Review and correction of draft manuscript.

**Conceptualization:** Min-Gwang Kim, Taek-Rim Yoon, Kyung-Soon Park.

**Data curation:** Min-Gwang Kim, Chae-Jin Im, Woo-Chul Jung.

**Formal analysis:** Min-Gwang Kim, Chae-Jin Im, Woo-Chul Jung.

**Investigation:** Min-Gwang Kim.

**Methodology:** Min-Gwang Kim.

**Project administration:** Min-Gwang Kim.

**Supervision:** Taek-Rim Yoon, Kyung-Soon Park.

**Validation:** Kyung-Soon Park.

**Visualization:** Kyung-Soon Park.

**Writing – original draft:** Min-Gwang Kim.

**Writing – review & editing:** Min-Gwang Kim.
